# Analysis of queries to a Swedish drug information centre identifies scientific knowledge gaps

**DOI:** 10.1038/s41598-024-82324-8

**Published:** 2024-12-06

**Authors:** Johan Nilsson, Jenny M. Kindblom, Julia Izsak

**Affiliations:** 1 Department of Drug Treatment, Region Västra Götaland, Sahlgrenska University Hospital, Gothenburg, Sweden; 2 Department of Psychiatry and Neurochemistry, Institute of Neuroscience and Physiology, The Sahlgrenska Academy, University of Gothenburg, Gothenburg, Sweden; 3 CTC Clinical Trial Consultants AB, Uppsala, Sweden; 4 Department of Internal Medicine and Clinical Nutrition, Institute of Medicine, The Sahlgrenska Academy, University of Gothenburg, Gothenburg, Sweden

**Keywords:** Evidence-based medicine, Clinical pharmacology, Drug information centre, Knowledge gap, Therapeutics, Drug therapy

## Abstract

Drug Information Centres (DICs) are providing clinicians with evidence-based support for rational drug treatment. However, knowledge gaps in the literature may hinder DICs from offering optimal guidance. This study examined the extent and nature of these knowledge gaps and their impact on clinical pharmacological advice, using real-world query data from a Swedish regional DIC. Data from 2022 at the Sahlgrenska University Hospital were analysed, focusing on queries outside off-label prescriptions and pharmaceutical aspects. A qualitative text content analysis identified phrases indicating a lack of evidence. Responses were categorized by the presence of these signalling phrases and the presence or absence of knowledge gaps. Among 209 responses, the majority were patient-specific (79%), posed by physicians (95%), and often related to adverse effects (37%). The analysis revealed that 23% of the responses had scientific knowledge gaps, and in 18% of the responses, no clinical pharmacological advice could be provided. Knowledge gaps were particularly associated with queries on adverse effects, long-term medication safety, specific patient cases and comorbidities, drug comparisons, or patient populations with limited literature coverage. This analysis highlights the potential of DICs to identify unmet clinical needs in drug treatment and to promote research for evidence-based, patient-centred drug treatment.

**Supplementary Information:**

The online version contains supplementary material available at 10.1038/s41598-024-82324-8.

## Introduction

Pharmacotherapy is a common and crucial instrument in clinical practice for treating diverse complex medical challenges. With the continuous introduction of novel therapies and the constantly growing body of literature, it becomes increasingly challenging for clinicians to navigate in the plethora of information. Drug Information Centres (DICs) are therefore important resources to support healthcare professionals^1^. DICs in Scandinavia are regional centres staffed by clinical pharmacologists and pharmacists that provide clinicians with objective, current, and evidence-based advice to guide them throughout the decision-making process in pharmacotherapy^1^. DICs address a broad spectrum of drug-related queries including polypharmacy, prevention of medication errors, addressing medication shortages, and investigating adverse drug reactions. They serve as a bridge between daily clinical practice and the advancements of the scientific community.

Despite the availability of numerous tools, such as Summaries of Product Characteristics (SmPC), decision support databases, textbooks, and medical databases, navigating the decision-making process in pharmacotherapy remains a challenging and time consuming task^2^. Retrieving, evaluating, and summarizing evidence requires a high level of expertise. This process has also been noted to face challenges due to knowledge gaps, which may further prolong response times at DICs^2,3^. Importantly, these knowledge gaps might hinder the provision of evidence-based responses, and in their absence, clinicians may resort to less well-founded practices. Thus, scientific knowledge gaps may potentially impact patient safety and treatment efficacy.

The frequency with which DICs encounter knowledge gaps within the literature is not well studied. A study conducted at a Brazilian DIC reports that roughly one-fourth of all drug-related queries lacked sufficient information in the consulted sources, resulting in inconclusive responses to clinicians^4^. Notably, over half of these inquiries pertained to off-label drug use (52%), such as alterations in pharmaceutical form or route of administration^4^, areas where information is often scarce. Given the large structural variations in healthcare systems in different countries, the challenges faced by clinicians are expected to vary considerably between sites. To the best of our knowledge, no such study has been conducted in Europe or in a Swedish setting.

This study aims to explore the query repertoire at a Swedish regional DIC and characterize the challenges faced by clinicians in everyday pharmacotherapy. By analysing real-world data from the DIC at Sahlgrenska University Hospital in Gothenburg during 2022 we intend to specifically examine to what extent information may be lacking in the literature, particularly within DIC responses containing comprehensive literature reviews. Furthermore, we aim to assess the potential implications of these scientific knowledge gaps for clinical pharmacological guidance. We envision that increasing awareness of the real-world challenges clinicians face in pharmacotherapy and addressing these knowledge gaps will lead to higher quality clinical guidance which may ultimately lead to improved pharmacotherapy.

## Methods

### Ethics

In this study we used anonymised publicly available responses from the regional DIC in Gothenburg, published on the national open-source database SVELIC (www.svelic.se). No ethical approval was required to conduct this study.

### The Drug Information Centre at the Department of Clinical Pharmacology, Sahlgrenska University Hospital

The DIC at Sahlgrenska University Hospital is a regional centre responsible for supporting healthcare workers in Region Västra Götaland. With a population of 1.7 million, constituting 17% of the Swedish population, the region is served by one university hospital, eight general hospitals, and around 200 primary healthcare centres. The DIC is operated by a team of specialist physicians, resident physicians, consultants, and pharmacists.

### The DIC standard operating procedures (SOP) at Sahlgrenska University Hospital

Queries to the DIC may be submitted by all healthcare professionals in the region, via telephone, e-mail, or by referral. The queries are logged in a local database and then distributed to an available investigator, i.e. resident physician or pharmacist. Each query is categorized into one or more relevant category such as adverse effects, documentation, drug interactions, pharmacokinetics, pregnancy and lactation, paediatric, or pharmaceutical aspects. The investigator conducts a comprehensive database and literature review, evaluates the results, and forms a response to the inquiring healthcare professional. Responses comprise relevant findings and in general include the following references: SmPCs, decision support databases (e.g., Lexicomp, UpToDate, Medicines Complete, Micromedex, Janusmed), textbooks (e.g., Meyler’s Side Effects of Drugs, Rang & Dale’s Pharmacology), as well as secondary and primary source medical databases (e.g., The National Institute for Health and Care Excellence (NICE), Cochrane Library, PubMed). The responses are discussed and approved by a senior physician (specialist or consultant in Clinical Pharmacology) before being finalized and sent to the inquiring healthcare professionals. As an additional quality assurance, all responses are internally reviewed at a weekly team meeting.

The responses are categorized in either comprehensive or short responses. Comprehensive responses are extensive and referenced reports that are formalized with a template and sent to the inquiring professional via letter, or e-mail. Comprehensive responses are further anonymised and uploaded to a national open-source database SVELIC for information dissemination. Short responses are typically less detailed, often due to factors such as time constraints, consistent findings from literature reviews that require minimal or no evaluation, or the presence of previous responses for similar queries. These short responses are communicated through phone calls or short e-mails.

### Data extraction procedure

In the present study, we extracted all queries managed by the DIC at Sahlgrenska University Hospital between 2022-01-01 and 2022-12-31. From the extracted data all non-relevant queries were excluded (i.e. referrals to other departments, queries not investigated for any reason). Queries with short responses were excluded as such responses do not imply a comprehensive literature search. Queries related to off-label treatment, and queries primarily related to pharmaceutical aspects such as manipulation of medicine (e.g., crushing), prescription, product characteristics, medication shortages and stability were further excluded. Additionally, responses that were not uploaded to SVELIC due to overly specific patient case descriptions were excluded from the analysis. In summary, our analysis focused on comprehensive responses that were outside off-label prescriptions and pharmaceutical aspects and were uploaded to the national open-source database SVELIC. This dataset represents a relevant sample of queries for examining the presence and extent of real-world clinical need-based knowledge gaps in the literature.

In addition to the actual written responses, the following parameters were extracted for each query: query category (adverse effects, documentation, drug interactions, pharmacokinetics, pregnancy and lactation, paediatrics), involved drugs and their Anatomical Therapeutic Chemical (ATC) classification codes, profession and clinic (hospital or primary care) of the inquiring healthcare professional, and the extent of query (patient specific or general).

In queries with multiple query categories, e.g. both a particular sub-population (such as paediatrics or pregnancy and lactation) and a type of drug query (such as adverse effects, documentation, drug interactions, or pharmacokinetics), queries were categorized based on population alone to ensure consistency and clarity. When queries could be categorized into several possible drug query types, a main category was selected to avoid duplicates in the analysis. A separate category was constructed for queries involving both adverse effects and interactions, as this combination represents a select group of queries with more complex nature.

ATC codes were retrieved from the Swedish *Farmacevtiska specialiteter i Sverige* (FASS), a national electronic medicines compendium that provides comprehensive information on drug products, including approved indications and classifications^5^. For drugs with multiple ATC codes, we used the registered product code based on trade name when available; if unavailable, classification was based on the primary therapeutic use within the clinical scenario presented in the patient query. All classifications were manually reviewed. In the analysis, we focused on level 1 ATC code categories representing major therapeutic groups.

### Text content analysis procedure

A qualitative text content analysis was completed manually in a three-step process. Step 1: Two observers (resident physicians, JN, JI) independently reviewed all responses identifying phrases, signalling lack of available studies. Step 2: From the responses with signalling phrases the observers identified cases where the response concluded that the evidence found was insufficient, indicating knowledge gaps. Step 3: From the responses with knowledge gaps the observers further identified responses including a clinical pharmacological guidance to the inquiring healthcare professional. Discrepancies between the independent observers (JN, JI) were resolved in a consensus discussion.

### Statistical analysis

Statistical analyses were conducted with Microsoft Excel (Microsoft 365).

## Results

In 2022, a total number of 618 queries were handled by our DIC, whereof 253 (40.9%) responses were comprehensive written responses, 301 responses (48.7%) were communicated by short responses via e-mail or phone calls, and 64 (10.4%) queries were categorized as non-relevant or were referred to another department.

### Qualitative text content analysis

Out of the 253 comprehensive written responses, a total of 44 were excluded from the analysis due to various reasons such as their focus on pharmaceutical aspects (*n* = 22), because they were not uploaded to SVELIC (*n* = 13) or were related to off-label prescriptions (*n* = 9).

**Fig. 1:**
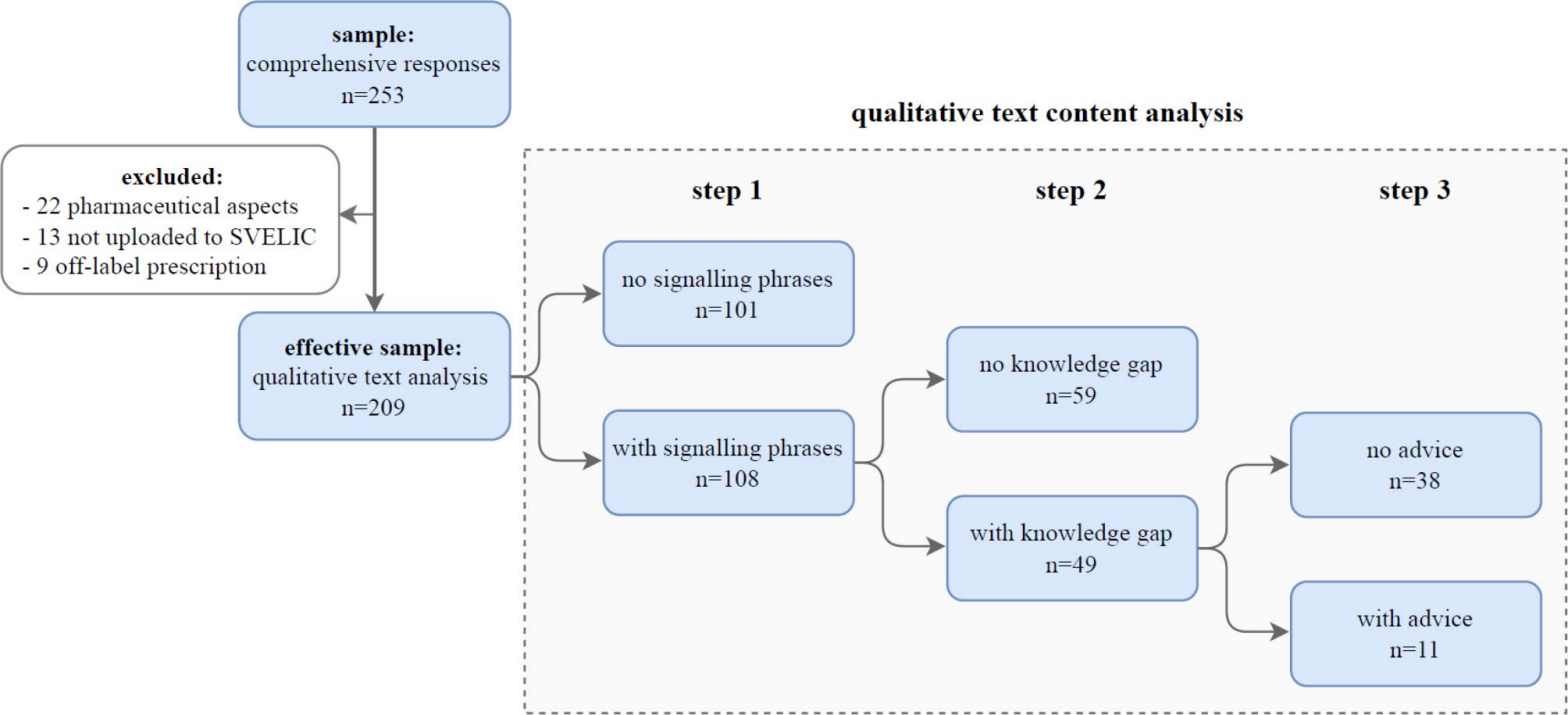
Flowchart of study sample and design. Schematic representation of the workflow and summary of qualitative text content analysis results.

For the remaining 209 responses a three-step qualitative analysis was conducted (Fig. 1).

In the first step, we assessed the responses with regard to content and phrases signalling a lack of evidence. Signalling phrases, such as “found no publication”, “studies are missing” or “weak evidence” were found in over half of the 209 responses (*n* = 108, 51.7%, Supplementary Table 1a).

In the second step, we conducted further text content analysis of the responses containing signalling phrases. Among them, 49 responses were identified as exhibiting scientific knowledge gaps, accounting for 45.4% (23.4% of all analysed responses). In the remaining 59 responses (54.6%), although containing signalling phrases, no knowledge gap could be identified. Thus, text analysis solely based on signalling phrases does not adequately capture scientific knowledge gaps.

In the third step, we categorized the selected 49 queries where knowledge gaps were identified, focusing on the presence of suggestions or advice. In 11 (22.4%) responses a suggestion was provided despite insufficient support in the literature. Among those, 6 of the responses provided suggestions based on available limited data and theoretical considerations. In 4 responses, advice was given for careful clinical follow-up of the patient and/or measurements of drug concentrations, while in 1 response, drug de-challenge was suggested (Supplementary Table 1b).

In the remaining 38 responses (77.6%), accounting for 18.2% of all analysed responses (*n* = 209), no clear suggestion or advice could be formulated (Fig. 1). Examples of drug queries with knowledge gaps and the provided advice are shown in Table 1, while all responses containing knowledge gaps are detailed in Supplementary Table 1b.

**Table 1:** Example queries with knowledge gaps and given advice. Table showing representative queries with knowledge gaps with and without a given advice.

Query	Knowledge gap	Given advice
Can abatacept be administered to patient with kidney failure?	No studies with abatacept in patients with kidney failure.	Caution is recommended, with careful monitoring.
What is described in the literature about treatment with vedolizumab in simultaneous malignancy?	No studies on treatment of patients with vedolizumab under ongoing cancer.	Extrapolates from studies and recommends careful follow-up.
Can PCSK9 inhibitors be used under ongoing treatment with etanercept?	No interaction studies with PCSK-9 inhibitors and etanercept -new drug.	Gives suggestion based on pharmacological properties.
What is known about the effect of baricitinib on male fertility?	No studies related to male fertility with use of baricitinib.	No advice
Is there anything described in the literature about the pharmacokinetic changes related to central stimulants after gastric bypass surgery?	No/limited study base for the effect of gastric bypass on pharmacokinetics of central stimulant drugs.	No advice
Are there any studies on the development of dementia with long-term use of solifenacin?	No studies on long-term effects of anticholinergics during young age and the development of dementia.	No advice

**Fig. 2:**
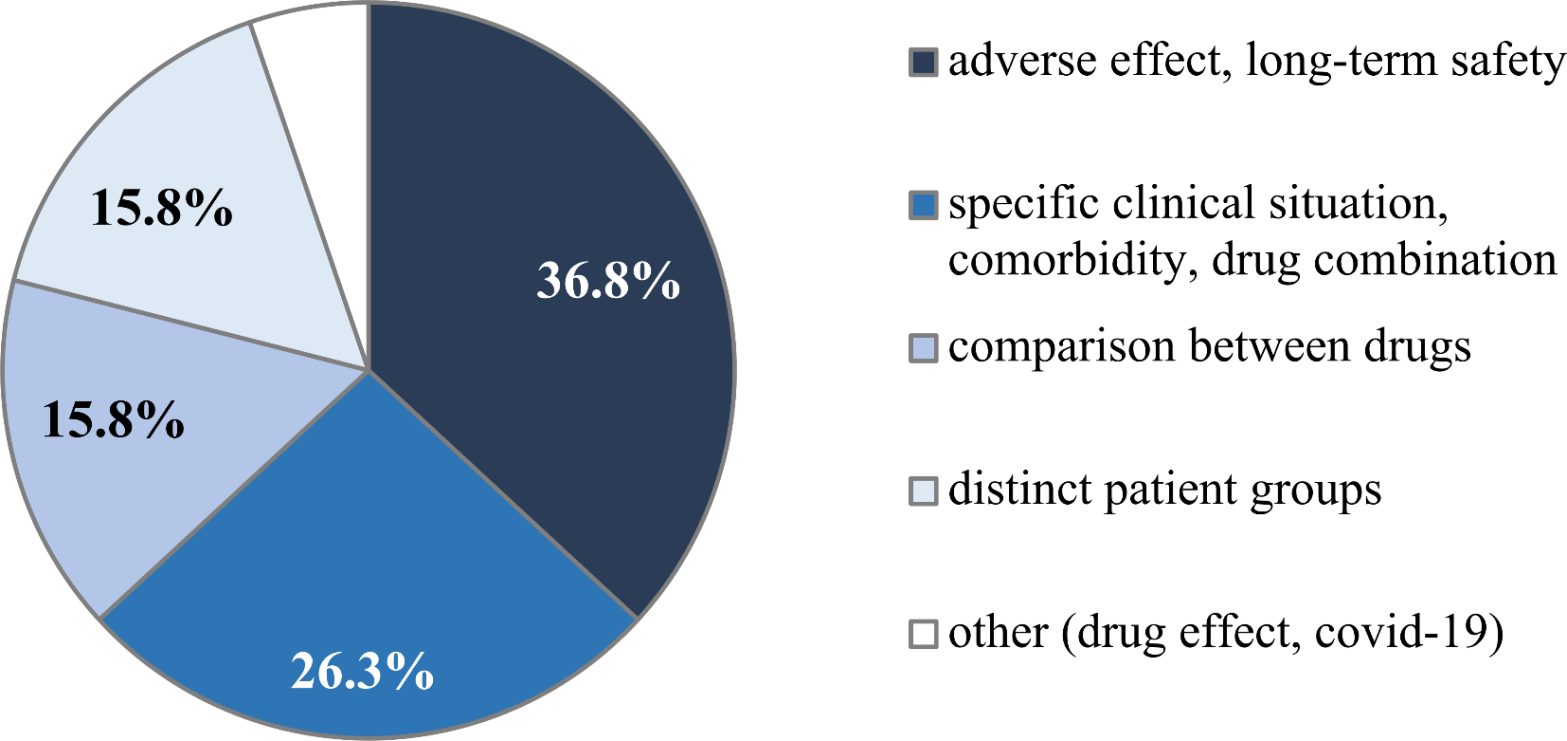
Queries lacking clinical pharmacological advice, categorized based on relevant areas of interest. Pie chart showing the distribution of topics in queries lacking clinical pharmacological advice.

Analysing the 38 responses lacking clinical pharmacological advice (Fig. 2), the majority focused on adverse effects or long-term safety of medications (*n* = 14, 36.8%), followed by queries related to specific clinical scenarios, unique comorbidities, or combinations of drugs (*n* = 10, 26.3%). Comparisons between drugs in terms of their effect or safety were also represented among inconclusive responses (*n* = 6, 15.8%). Additionally, queries focusing on distinct patient groups, such as pregnant and lactating women, post-bariatric surgery patients, and children, were notable as well (*n* = 6, 15.8%).

### Queries and categories

Most responses (78.5%) were related to specific patient cases, primarily from hospital healthcare workers (82.3%), and most inquiries were made by medical doctors (94.7%). A similar distribution was observed even in queries with knowledge gaps and in those without a given advice (Table 2).

**Table 2:** Query descriptives. Table showing descriptives of all analysed responses, responses with knowledge gaps and responses with no given advice.

	All responses *n* = 209 (%)	Knowledge gaps *n* = 49 (%)	No advice *n* = 38 (%)
Extent of query			
Patient specific	164 (78.5)	38 (77.6)	27 (71.1)
General	45 (21.5)	11 (22.4)	11 (28.9)
Point of care			
Primary care	37 (17.7)	6 (12.2)	6 (15.8)
Hospital	172 (82.3)	43 (87.8)	32 (84.2)
Inquiring professional			
Medical doctor	198 (94.7)	47 (95.9)	36 (94.7)
Pharmacist	8 (3.8)	1 (2.0)	1 (2.6)
Nurse	3 (1.4)	1 (2.0)	1 (2.6)

Among the 209 published responses, the three most frequent query types were adverse drug reactions (*n* = 78, 37.3%), drug interactions (*n* = 48, 23%), and documentation and evidence behind pharmacotherapy (*n* = 46, 22%). These categories remained prominent even in queries with knowledge gaps and those with inconclusive responses (Table 3). Specifically, adverse drug reactions and documentation were highly represented across all response types, with 36.7% and 30.6% occurrence in queries with knowledge gaps, respectively, and 39.5% and 26.3% occurrence in responses with no advice. In contrast, queries related to drug interactions were less prominent in both queries with knowledge gaps (14.3%) and responses with no advice (10.5%).

**Table 3:** Query categories. Table summarizing query categories in all analysed responses, in responses with knowledge gaps and in responses with no given advice.

Query category	All responses *n* = 209 (%)	Knowledge gaps *n* = 49 (%)	No advice *n* = 38 (%)
Adverse effects	78 (37.3)	18 (36.7)	15 (39.5)
Drug interactions	48 (23.0)	7 (14.3)	4 (10.5)
Documentation	46 (22.0)	15 (30.6)	10 (26.3)
Pregnancy and lactation	16 (7.7)	4 (8.2)	4 (10.5)
Pharmacokinetics	8 (3.8)	2 (4.1)	2 (5.3)
Paediatrics	7 (3.3)	1 (2.0)	1 (2.6)
Adverse effects and interactions	5 (2.4)	2 (4.1)	2 (5.3)
Other	1 (0.5)	0 (0)	0 (0)

### Analysis of ATC-codes

The analysed 209 queries involved in total 458 ATC code entries. Among these, 49 queries with knowledge gaps comprised 94 drug entries, while the 38 responses without advice included 72 records. Of note, one query might include several drugs, and the same drug can occur in more than one query. The majority of ATC code entries accounted for drugs related to the nervous system, *n* = 118 (25.8%), followed by antineoplastics and immunomodulating agents (*n* = 97, 21.2%) and drugs related to the cardiovascular system (*n* = 40, 8.7%) (Table 4). Examining responses with knowledge gaps, drugs targeting the nervous system were still prevalent, comprising 26.6% (*n* = 25) of entries, while antineoplastic and immunomodulating agents represented 33.0% (*n* = 31). In responses with no advice, drugs targeting the nervous system remained prominent at 30.6% (*n* = 22), followed by antineoplastics and immunomodulating agents at 29.2% (*n* = 21). Interestingly, drugs related to the cardiovascular system were less represented in both categories, with only 3.2% (*n* = 3) in responses with knowledge gaps and 2.8% (*n* = 2) in responses with no advice (Table 4).

**Table 4:** ATC code entries. Table describing ATC code entries in all analysed responses, in responses with knowledge gaps and in responses with no given advice.

ATC code groups	All responses *n* = 458 (%)	Knowledge gaps *n* = 94 (%)	No advice *n* = 72 (%)
Nervous system (N01-N07)	118 (25.8)	25 (26.6)	22 (30.6)
Antineoplastic and immunomodulating (L01-L04)	97 (21.2)	31 (33.0)	21 (29.2)
Cardiovascular system (C01-C10)	40 (8.7)	3 (3.2)	2 (2.8)
Blood and blood forming organs (B01-B06)	38 (8.3)	5 (5.3)	4 (5.6)
Antiinfectives for systemic use (J01-J07)	36 (7.9)	10 (10.6)	5 (6.9)
Genito urinary system and sex hormones (G01-G04)	31 (6.8)	7 (7.4)	5 (6.9)
Alimentary tract and metabolism (A01-A16)	28 (6.1)	1 (1.1)	1 (1.4)
Musculo-skeletal system (M01-M09)	25 (5.5)	5 (5.3)	5 (6.9)
Respiratory system (R01-R07)	17 (3.7)	4 (4.3)	4 (5.6)
Dermatologicals (D01-D11)	11 (2.4)	3 (3.2)	3 (4.2)
Systemic hormonal preparation (H01-H05)	11 (2.4)	0 (0)	0 (0)
Various (V01-V10)	3 (0.7)	0 (0)	0 (0)
Antiparasitic product (P01-P03)	2 (0.4)	0 (0)	0 (0)
Other	1 (0.2)	0 (0)	0 (0)

## Discussion

In this study, using real-world data from a regional DIC, we observed scientific knowledge gaps in the literature for 49 out of 209 queries concerning pharmacotherapy encountered in everyday clinical practice. Our study supports previous reports indicating a gap between the knowledge demanded by clinical practice and patient-centred work versus the research conducted in academia^6,7^. We found that almost one fourth (23.4%) of all the responses to relevant drug-related queries from healthcare professionals lacked sufficient supporting evidence in the medical literature. In the majority (77.6%) of responses with knowledge gaps, accounting for nearly one fifth (18.2%) of the total analysed responses, no clinical pharmacological advice could be formulated to the inquiring professional.

Research on the extent of knowledge gaps in real-world clinical situations is scarce; however, there are a few studies that have explored this topic, particularly for certain types of queries. Although our rate of queries with inconclusive responses aligns with previous studies^4,8–10^, there are some discrepancies that warrant further discussion. For instance, in a study from Brazil, a quarter (25%) of the responses showed insufficient support in the literature. However, over half of the responses, where lack of information was identified, was related to off-label drug use^4^. In our study, responses that relied on empirical experience and off-label prescriptions were deliberately excluded. The results in this study suggest, therefore, a higher overall prevalence of identified knowledge gaps, despite methodological differences. Further, previous studies addressing drug queries related to pregnancy reported conflicting results regarding information availability in the literature. In line with our findings where 25% (4/16) of the queries related to pregnancy and lactation lacked a conclusive advice, a DIC study from the United States reported that 27% of the answers provided to callers during 2006–2010 were limited due to lack of information availability^8^. A study from a Norwegian DIC that analysed queries related to drug safety during pregnancy reported that all the analysed responses provided some information to the inquiring healthcare professionals, suggesting a low proportion of knowledge gaps^9^. However, in the present study, even though some information was available in the literature, it was often conflicting and considered insufficient to offer adequate guidance to the clinician. Additionally, a previous analysis from our regional DIC at Sahlgrenska University Hospital that focused on queries related to drug interactions addressed between 2008 and 2017, reported that 95% of the analysed queries received an advice^10^. This mirrors our findings and suggests that the proportion of queries related to drug interactions with a provided advice did not change over time. Nevertheless, the high proportion of queries related to drug interactions highlights the need for consistent and reliable drug interaction information for clinicians. Previous studies have shown significant variability across drug information resources, especially in interaction severity and clinical recommendations, which can pose risks to patient safety^11,12^. This inconsistency emphasizes the importance of standardizing and harmonizing drug interaction documentation to support safer clinical decision-making.

Although this study did not analyse short answers, it is worth noting that approximately half of the queries responded to by the DIC were communicated via short answers. This observation may have several explanations. One explanation may be related to time constraints on DIC staff or clinicians, particularly when prompt responses are required. Other factors, such as limited access for clinicians to certain databases, suboptimal user-friendliness of decision-support tools, and gaps in clinicians’ search strategy training, may further explain the proportion of shorter responses. Addressing these areas through improvements in database design and raising awareness of available support tools among clinicians could enhance information accessibility.

In this study the majority of the inquiring healthcare professionals were medical doctors (94.7%), predominantly working in hospital settings (82.3%). This profile highlights the complex and specialized nature of the received queries. Notably, responses indicating knowledge gaps were more frequent among queries from clinicians working in hospital settings (87.8%). This trend may be attributed to the likelihood that queries are addressed to the DIC only after initial consultation with colleagues, particularly in challenging or atypical cases of highly specialized care. Such filtering process suggests that the queries reaching the DIC are highly clinically relevant and may require further specialized expertise.

Occasions where knowledge gaps in pharmacotherapy resulted in the inability to provide advice were predominantly linked to adverse effects and long-term safety of medication use in the present study. Particularly notable was the high frequency of uncertainties observed with nervous system-related drugs. Despite their common usage, there is often a lack of extensive research evidence regarding the prolonged use of psychiatric drugs^13^. Additionally, novel therapies within ATC codes linked to antineoplastics and immunomodulating agents, dermatologicals, as well as respiratory system drugs, were also associated with knowledge gaps and inconclusive responses. This may be attributed to the scarcity of literature and the limited empirical experience in this rapidly evolving field, contributing to the observed uncertainties in everyday clinical practice.

Queries related to drug comparisons in terms of both efficacy and safety were also frequently encountered among responses with uncertain conclusion. Our findings align with the recognized importance of prioritizing comparative-effectiveness studies to determine the interventions that are most effective for specific patient types^14^. Queries focusing on distinct patient groups, such as pregnant and lactating women, post-bariatric surgery patients, and children, were also noteworthy. These patient groups where drug pharmacokinetics may be less predictable or altered are often excluded from clinical trials and underrepresented in epidemiological research^15–17^. Despite receiving limited attention in research, these patient groups constitute an important portion of everyday clinical situations, as indicated by our findings.

Taken together, our results identify relevant areas with knowledge gaps using real-world clinical data. Consequently, these findings highlight the potential for the DIC to serve as a valuable tool in identifying clinical need-based knowledge gaps in the future. Identifying knowledge gaps is a recognized important mission for the Swedish Agency for Health Technology Assessment that actively engage in collecting knowledge gaps and evidence uncertainties in order to identify and prioritize areas where new primary research is needed^18^. Aligning with these national initiatives and positioning DICs as resources for detecting signals of knowledge gaps could set the framework for establishing a new knowledge gap inventory grounded in real-world clinical needs. Future efforts implementing standardised and automatized procedures including artificial intelligence for the continuous identification of these knowledge gaps would be highly beneficial. An established inventory with real-world knowledge gaps has the potential to promote research initiatives driven by patient needs. This could contribute to bridging the gap between clinical need and academic research and pave the way to better and safer pharmacotherapy for our patients.

The study has some limitations that warrant further consideration. Firstly, the text analysis performed by the authors introduces a potential for subjectivity in the data. The objective method used to identify responses with knowledge gaps, relying on the presence of signalling words, proved to be overly sensitive and necessitated further subjective assessment. To reduce subjectivity, all responses underwent analysis by two independent observers (authors JN, JI), and any discrepancies were resolved through a consensus discussion. For further transparency, all queries lacking sufficient support are documented in the supplementary material. This manual review process was time-consuming. To improve efficiency in future studies, refining the signalling words used to detect knowledge gaps could help reduce the manual workload. Excluding terms that frequently appear in responses without knowledge gaps may make the process more focused and improve the accuracy of the initial flagging system. In addition, incorporating automated tools, such as natural language processing and artificial intelligence, could provide valuable support in identifying language patterns commonly associated with scientific knowledge gaps. While artificial intelligence tools do not inherently ensure objectivity, they could significantly reduce manual time-consuming work effort and improve consistency in identifying knowledge gaps. Additionally, although artificial intelligence cannot replace human judgement, it could assist DICs in their workflow by linking queries to relevant literature and utilizing past queries and responses, thereby enabling faster and more accurate answers while minimizing human intervention.

A limitation related to challenges in categorizing complex queries is also worth mentioning. Many queries involve multiple aspects, making it difficult to fit them into a single category. While we aimed for consistency by focusing on the primary patient population or drug-related issue, this approach may not have fully captured the complexity of some queries. Further, queries classified as lacking sufficient evidence in the literature may stem from time constraints during investigation, and we cannot entirely exclude potential subjective bias or errors in the analysed responses. However, the SOP of the DIC incorporates several quality safeguards including review by a board-certified specialist in clinical pharmacology and weekly joint conferences allowing for a second review. Lastly, while our study revealed a considerable number of drug-related queries with knowledge gaps, potentially resulting in insufficient guidance for clinicians, predicting the actual consequences on rational drug use and patient care remains challenging. It is rather difficult to measure the consequence of these knowledge gaps on decision making given the typical low response rates in clinical survey studies^19^. Future efforts are needed to map the extent of consequences of the existing knowledge gaps on the health care of patients.

## Conclusion

Analysis of drug-related queries from a Swedish regional DIC highlights the presence of scientific knowledge gaps, potentially leading to inadequate guidance for clinicians. Consequently, DICs may be important resources for identifying unmet needs from everyday clinical practice, promoting future research for evidence-based medicine and patient-centred drug treatment.

## Electronic supplementary material

Below is the link to the electronic supplementary material.

Supplementary Material 1

## Data Availability

The raw data used in this study are available on the open-source database www.svelic.se.
