# Author Correction: Analysis of queries to a Swedish drug information centre identifies scientific knowledge gaps

**DOI:** 10.1038/s41598-025-93050-0

**Published:** 2025-03-27

**Authors:** Johan Nilsson, Jenny M. Kindblom, Julia Izsak

**Affiliations:** 1 Department of Drug Treatment, Region Västra Götaland, Sahlgrenska University Hospital, Gothenburg, Sweden; 2 Department of Psychiatry and Neurochemistry, Institute of Neuroscience and Physiology, The Sahlgrenska Academy, University of Gothenburg, Gothenburg, Sweden; 3 CTC Clinical Trial Consultants AB, Uppsala, Sweden; 4 Department of Internal Medicine and Clinical Nutrition, Institute of Medicine, The Sahlgrenska Academy, University of Gothenburg, Gothenburg, Sweden

Correction to: *Scientific Reports* 10.1038/s41598-024-82324-8, published online 06 December 2024

The original version of this Article contained an error in Methods. The subheading ‘Verification of the method of compiling available evidence at the DIC in Gothenburg’ referred to an internal check within the Drug Information Centre (DIC), rather than a formal method used in the current study. Since this information did not influence the article’s results or conclusions, the text below and reference 6 were removed.

“Prior to the start of this project, an internal analysis was conducted in cooperation with the regional Health Technology Assessment (HTA) centre to verify our method of summarizing the available evidence. The HTA centre is a regional support organisation that conducts systematic literature assessments with the help of well-established standardised templates and GRADE system approach for investigating methods and interventions within the health care system^6^. Two clinical queries were investigated by both the HTA centre and the regional DIC, independently of each other, using separate standard operating procedures (SOPs). No major discrepancies were identified in the conclusion of the two investigations (unpublished data, data not shown).”

The original Article has been corrected.

