# Supplementary material for: Analysis of queries to a Swedish drug information centre identifies scientific knowledge gaps
Source: Sci Rep. 2024 Dec 6;14:30412. doi: 10.1038/s41598-024-82324-8 (PMC11621409; doi:10.1038/s41598-024-82324-8)
Supplement: Supplementary file 1 — Supplementary Material 1 [file 41598_2024_82324_MOESM1_ESM.pdf]

# Analysis of queries to a Swedish Drug Information Centre identifies scientific knowledge gaps

Johan Nilsson, Jenny M. Kindblom, Julia Izsak

Signalling phrases from the 49 responses with knowledge gaps were extracted and categorized into two main types: phrases signalling missing studies or data and phrases suggesting limited data or uncertain evidence. Phrases signalling missing studies or data were present 62 times, while phrases suggestive of limited available data were present 50 times in these responses (Supplementary Table 1a).

**Supplementary Table 1a:** Summary of signalling phrases extracted from queries with identified knowledge gaps

| **Missing studies/data** | **No** (n=62) | **Limited data** | **No** (n=50) |
|---|---|---|---|
| found no publication/data/information | 31 | limited data/documentation/knowledge | 19 |
| missing studies/data/documentation | 28 | a few studies/case reports | 11 |
| not have been investigated | 2 | weak/lacking evidence/support | 11 |
| missing standardised methods | 1 | insufficiently/poorly/sparsely studied | 9 |

**Supplementary Table 1b:** Queries with knowledge gaps and given advice.

| No. | Query | Knowledge Gap | Given advice |
|---|---|---|---|
| **1.** | Is there a clinically relevant interaction between remdesivir and the patient's other medication? | No interaction studies available between remdesivir and other drugs. | Recommends careful follow-up and drug concentration measurements. |
| **2.** | Can a patient who has experienced anaphylaxis from the BRAF/MEK inhibitor combination dabrafenib/trametinib use encorafenib/binimetinib? | No cross-reactivity studies between different BRAF/MEK inhibitor combinations. | Recommends careful follow-up. |
| **3.** | Can abatacept be administered to patient with kidney failure? | No studies with abatacept in patients with kidney failure. | Caution is recommended, with careful monitoring. |
| **4.** | COVID-19 vaccine after a bleeding reaction to Moderna's vaccine in a patient with allogeneic stem cell transplantation - what would you recommend? | No information in the literature related to bleeding complications of Covid vaccine in patients after allogenic stem cell transplantation. | Gives suggestion based on product characteristics. |
| **5.** | Can Nexplanon be combined with lamotrigine? | No interaction studies available between Nexplanon and lamotrigine. | Recommends careful follow-up and drug concentration measurements. |
| **6.** | What is described in the literature about treatment with vedolizumab in simultaneous malignancy? | No studies on treatment of patients with vedolizumab under ongoing cancer. | Extrapolates from studies and recommends careful follow-up. |
| **7.** | Can a patient with antithrombin deficiency be treated with dapsone? | No data on treatment with dapsone in patient with lack of antithrombin. | Gives suggestion based on a theoretical discussion. |
| **8.** | Use of combined oral contraceptives for patients with discoid lupus erythematosus. What is known? | No/limited data on use of combined contraceptives in patients with discoid lupus. | Gives suggestion based on a theoretical discussion. |
| **9.** | What is the recommended maximum dose, dosing interval, and treatment duration when using lidocaine spray? | No/limited data on max dose and exposure period for topical lidocaine. | Extrapolates from available data. |
| **10.** | Is leg pain a known side effect of pregabalin? How long does the side effect persist? | No data on how long the side effect can be expected to remain. | Gives suggestion of de-challenge. |
| **11.** | Can PCSK9 inhibitors be used under ongoing treatment with etanercept? | No interaction studies with PCSK-9 inhibitors and etanercept -new drug. | Gives suggestion based on pharmacological properties. |
| **12.** | Are there current studies regarding the use of topical hydrocortisone for umbilical granuloma? | No studies on the effect and safety of topical mild steroids for the treatment of umbilical granuloma. | - |
| **13.** | Can Pandemrix vaccine cause side effects such as hypersomnia or parasomnia? | No studies/evidence related to hypersomnia or parasomnia in relation to Pandemrix vaccine. | - |
| **14.** | Can guselkumab worsen prostate cancer? | No data for a new IL-23 inhibitor and the risk for worsening prostate cancer/cancer. | - |

| No. | Query | Knowledge Gap | Given advice |
|---|---|---|---|
| **15.** | What is known regarding side effects during long-term treatment with sertraline? | No studies related to long-term treatment with sertraline and adverse effects. | - |
| **16.** | Can high-dose escitalopram over an extended period lead to permanent damage? | No studies related to long-term treatment and safety with high dose escitalopram. | - |
| **17.** | Is there evidence of a causal relationship between pembrolizumab and genital issues? | No studies or reports on side effects of pembrolizumab on vaginal epithelium. | - |
| **18.** | What are the risks of long-term treatment with the combination drug containing cinnarizine and dimenhydrinate? | No studies on long-term treatment with the combination drug containing cinnarizine and dimenhydrinate. | - |
| **19.** | The risk profile of psychiatric medications during pregnancy and lactation. | No- or limited evidence related to psychiatric medications during pregnancy and lactation. | - |
| **20.** | What is known about the effect of baricitinib on male fertility? | No studies related to male fertility with use of baricitinib. | - |
| **20.** | Is there anything described in the literature about concurrent treatment with omalizumab and cyclosporine? | No DDI studies with omalizumab and cyclosporine. | - |
| **22.** | Pseudoporphyria triggered by naproxen - is etoricoxib a safe alternative? | No studies related to risk for pseudoporphyiria with different COX-inhibitors. | - |
| **23.** | Is there anything described in the literature about the pharmacokinetic changes related to central stimulants after gastric bypass surgery? | No/limited study base for the effect of gastric bypass on pharmacokinetics of central stimulant drugs. | - |
| **24.** | Can a patient who developed reactive arthritis after a Covid-19 infection be vaccinated with the third dose of the vaccine without the risk of reactivation? | No data related revaccination of patient that developed reactive arthritis after covid infection. | - |
| **25.** | What documentation is available on atorvastatin-induced alopecia areata? Can atorvastatin be reintroduced, or should one choose another drug? | Alopecia areata as a known side effect of statins. No studies/comparative studies available. | - |
| **26.** | Can beta-blockers trigger lichen planus? | Limited data on lichen planus and beta-blockers. | - |
| **27.** | Is there an interaction between methotrexate and natalizumab, dimethyl fumarate, or cladribine? Is there any combination that is particularly vulnerable? | No data on combined treatment with dimethyl fumarate and methotrexate. | - |

| No. | Query | Knowledge Gap | Given advice |
|---|---|---|---|
| **28.** | Does the risk of developing progressive multifocal leukoencephalopathy differ between natalizumab, cladribine, and dimethyl fumarate? | No studies that compare the risk of progressive multifocal leukoencephalopathy between dimethyl fumarate, cladribine, natalizumab. | - |
| **29.** | Are there any case reports where adalimumab has triggered cutaneous lupus erythematosus? | Limited data related to the relation between adalimumab and cutaneous lupus erythematosus. | - |
| **30.** | Can levetiracetam or topiramate affect egg maturation or the breakdown of choriogonadotropin alfa? | No/limited study material on epilepsy medicine and effects in IVF/fertility. | - |
| **31.** | Is there evidence that norethisterone is more effective or has fewer side effects compared to medroxyprogesterone in menstrual cycle regulation? | No studies comparing the effect of risk of norethisterone and medroxyprogesterone for menstrual cycle regulation. | - |
| **32.** | Are migrating myalgia and fatigue possible long-term side effects of isotretinoin? | No/limited studies on long-term adverse effects of isotretinoin in form of myalgia and fatigue. | - |
| **33.** | What is the evidence behind cough medicines, with focus on pentoxiverine? | No studies/evidence behind use of pentoxiverine as cough medicine. | - |
| **34.** | Are there risks associated with using lurasidone during pregnancy? | No/limited studies on lurasidone during pregnancy. | - |
| **35.** | Can codeine be given to a patient with a previous severe allergic reaction to ketobemidone? | Limited data on opioid cross reactivity in patients with allergy. | - |
| **36.** | Interaction risk between cabozantinib and apixaban? Is edoxaban a more suitable alternative? | Limited data on interaction risks with NOAK and cabozantinib. | - |
| **37.** | Does the absorption of venlafaxine and mirtazapine change after gastric bypass surgery? | Limited data on venlafaxine and mirtazapine in patients after gastric bypass. | - |
| **38.** | How established is the link between SSRI or SNRI and microscopic colitis? | No clear evidence behind the causality of SSRI/SNRI and microscopic colitis. | - |
| **39.** | Is there any association between exposure to acetylsalicylic acid during pregnancy and hearing impairment in the child? | Low evidence behind acetylsalicylic acid exposure during pregnancy and its ototoxicity on offspring. | - |
| **40.** | Are there any studies on the development of dementia with long-term use of solifenacin? | No studies on long-term effects of anticholinergics during young age and the development of dementia. | - |
| **41.** | What is described regarding the interaction between acitretin and intermittent treatment with doxycycline? | No/limited data for combination of doxycycline and acitretin and the risk for intracranial hypertension. | - |

| No. | Query | Knowledge Gap | Given advice |
|---|---|---|---|
| **42.** | Bleeding risk with simultaneous treatment of COX-2 inhibitors and dalteparin? | No data to compare the risk for bleeding with different COX-inhibitors in combination with dalteparin. | - |
| **43.** | Are there any case reports of potential worsening of T-cell lymphoma/skin lymphoma in connection with treatment with guselkumab and risankizumab? | Limited data on safety of guselkumab and risankizumab in patients with T-cell/skin lymphoma. | - |
| **44.** | Is there documentation for the long-term use of propiomazine and promethazine as sleep medication? | No studies on long-term treatment with propiomazine and promethazine for sleep. | - |
| **45.** | Genetic component in drug-induced neutropenia? | Limited studies on genetical predisposition for drug induced neutropenia. | - |
| **46.** | Are there published case reports of combination therapy with vedolizumab and etanercept? | Limited data on interaction risks with vedolizumab and etanercept. | - |
| **47.** | Is cross reactivity described between adalimumab and etanercept? | No studies on cross reactivity between adalimumab and etanercept. | - |
| **48.** | Can orphenadrine and chlorzoxazone be considered equivalent in terms of efficacy and safety? | Limited evidence related to effect and safety of chlorzoxazone compared to orphenadrine. | - |
| **49.** | Does aripiprazole impair the effectiveness of zuclopenthixol? | Limited studies related to combination of aripiprazole and zuclopenthixol. | - |
